# Role of Follow-up Radiographs After Pin Removal in Distal Humeral Fractures in Children: An Ambi-Directional Cohort Study

**DOI:** 10.7759/cureus.96318

**Published:** 2025-11-07

**Authors:** Sireethorn Chatpermporn, Pipattra Sailohit

**Affiliations:** 1 Orthopaedics, Police General Hospital, Bangkok, THA

**Keywords:** baumann angle, elbow injuries, lateral condylar fracture, pediatric distal humerus fracture, supracondylar fracture humerus

## Abstract

Background: Distal humeral fractures are the most common pediatric elbow fractures. Although frequent, displaced fractures are still challenging in treatment and may lead to unfavorable elbow deformities as long-term complications. Post-surgical evaluation always includes plain radiographs for evaluating alignment and bone union. However, there is currently no evidence supporting the role of follow-up radiographs at different time periods after pin removal.

Objectives: This study aimed to compare the Baumann angle and lateral humeral-capitellar angle (LHCA) at pin-removal day, short-time period (1-2 weeks after removal), and long-time period (>12 weeks after removal).

Study design: The study includes an ambi-directional cohort.

Settings: The study was conducted in the Police General Hospital.

Subjects: Pediatric patients (<14 years old) with distal humeral fractures who underwent pinning were included in the study.

Methods: This study was conducted on 137 patients from the radiograph database from 2010 to 2024. Plain radiographs of the elbow in AP and lateral views were used for measuring the Baumann angle and LHCA. The calculation was performed using IBM SPSS Statistics for Windows, Version 16 (Released 2015; IBM Corp., Armonk, New York, United States).

Results: The Baumann angles at the pin-removal day, short-time period, and long-time period were 71.95^o^ ± 3.77^ o^, 71.92^ o^ ± 3.84^ o^, and 72.12^ o^ ± 3.76^ o^, respectively. The LHCA was 51.44 ± 2.29, 51.37 ± 2.47, and 50.92 ± 3.42, respectively. Statistical analysis results show no significant difference in each period for the Baumann angle (p=0.368), but a slightly significant decrease in the LHCA at the long-time period (p=0.01). Subgroup analysis was performed, indicating no difference in the Baumann angle and LHCA among age groups (2-5 years old vs >5 years old). For subgroup analysis of fracture locations, the lateral condylar humerus fracture group had a significant change in the LHCA about 3.25^o^ at a long-time period (p=0.043). However, the angles were all in the normal range.

Conclusions: Follow-up plain radiographs demonstrated that the Baumann angle remained stable across the pin-removal day, short-term, and long-term, regardless of age or fracture location. In contrast, the LHCA showed a significant but small decrease at long-term follow-up, particularly in patients with lateral condylar fractures, suggesting a remodeling tendency specific to this subgroup. These could indicate no role of follow-up radiographs after pin removal.

## Introduction

Distal humeral fractures represent one of the most common pediatric injuries, accounting for approximately 16% of all fractures in children [1-5]. These include supracondylar fractures (55-75%), followed by lateral condylar fractures (15%) and medial condylar fractures (5%) [6]. Despite the frequency, displaced pediatric distal humeral fractures remain challenging owing to the complexity of elbow development and the requirement for surgical management [7,8].

Closed reduction and percutaneous pinning are the standard treatment for displaced pediatric distal humeral fractures [9-12]. Open reduction and pinning are generally reserved for cases of significant displacement, resulting in instability, such as supracondylar fractures (Modified Gartland Type 4), lateral condylar fractures (Song Stage 5), or cases with non-anatomic reduction after closed reduction and pinning [13,14]. Post-operative complications, including neurological and vascular injury, pin-tract infection, avascular necrosis, nonunion, loss of reduction and cubitus varus/valgus deformity resulting from malunion or nonunion, are major clinical concerns [12,15-19]. Cubitus varus deformity usually is a complication presented in patients with malunion supracondylar fractures or lateral condylar fractures [12]. Cubitus valgus deformity may be present in patients with nonunion lateral condylar fractures (nonunion after 12 weeks with subsequent displacement) [12]. Medial epicondylar fractures, though rare, could also lead to cubitus valgus [20].

Post-operative management typically involves serial radiographs to assess bone union and alignment. Pin and cast removal are generally performed after 3-6 weeks. However, there are currently no established guidelines regarding the optimal timing or duration of radiographic follow-up after the pin removal [21-23].

The Baumann angle is formed by the humeral axis and a straight line through the epiphyseal plate of the capitellum. Measurement is done in anteroposterior radiographs with the elbow in extension [24]. An increase in the Baumann angle will lead to cubitus varus and internal rotation deformities [24,25].

The lateral humeral-capitellar angle (LHCA) is formed by the anterior border of the distal humeral shaft and a line along the capitellar physis. Measurement is done on the lateral radiographs with the elbow in 90-degree flexion. A change in the LHCA depicts angular deformities [26,27]. 

This study aimed to evaluate the role of follow-up radiographs after pin removal at different intervals. Specifically, the study investigated changes in radiographic parameters associated with elbow deformity, with the goal of informing post-operative management strategies in displaced pediatric distal humeral fractures.

## Materials and methods

In this ambi-directional cohort study, we studied all pediatric patients diagnosed and treated at the Police General Hospital from January 2010 to January 2025. Retrospective data (2010-2022) were retrieved from the database. Prospective data were collected from patients visiting beyond November 2023. Eligibility criteria included: 1) Patients with displaced supracondylar humeral fracture, lateral condylar humeral fracture or medial epicondylar humeral fracture; 2) Patients younger than 14 years old; 3) Patients who underwent closed or open reduction and pinning for treatment and 4) Patients who had radiographs at pin removal day, 1-2 weeks and beyond 12 weeks. Patients were excluded if they met any of the following criteria: 1) Patients with a congenital deformity at elbow; 2) Patients with a suspected diagnosis of pathological fractures or 3) Patients with prior or concurrent ipsilateral upper extremity fractures.

Depending on the patient’s fracture displacement and stability, the surgeons decided to perform either closed or open reduction. Two or three Kirschner wires were used for pinning. All patients had intraoperative fluoroscopy confirming adequate reduction before the long arm cast was applied. Adequate radiographs in anteroposterior view and lateral view were performed in all patients in the prospective group.

Radiographs were reviewed and measured for the Baumann angle, LHCA, anterior humeral line alignment and number of cortices with callus by one pediatric orthopedist. Demographic data and total days of pinning were collected through electronic medical records.

The Baumann angle and LHCA on the pin removal day, 1-2 weeks and beyond 12 weeks were compared with a repeated measure ANOVA test. Anterior humeral line alignment and the number of cortices with callus at different intervals were compared with McNemar’s test and Cochran’s Q test, respectively. Quantitative parameters were expressed as the mean value with standard deviation. The significance level was set at 0.05. The data were analyzed with STATA version 16 (StataCorp LLC, College Station, Texas, USA).

This study received approval from the Institutional Review Board of Police General Hospital and consent was obtained from all patients. Assent forms were obtained from parents or representatives.

## Results

A total of 137 patients were included in this study. Patient age ranged from 1 to 13 years old (mean age 5.78 ± 2.8). The mean duration of pinning was 37.9 ± 3.38 days. Of 137 patients, 83 (60.6%) were male. Regarding the fracture location, supracondylar humeral fractures, lateral condylar humeral fractures and medial epicondylar humeral fractures were 53.3%, 39.4% and 7.3% respectively. The majority of patients (73.7%) were treated with closed reduction (Table 1).

**Table 1:** Clinical characteristics (n=137)

Parameters	N (%)	p-value
			Age at diagnosis (years)		0.18
1-5 (cases)	71 (51.8)				
>5 (cases)	66 (48.2)				
Gender		0.013
Male	83 (60.6)	
Female	54 (39.4)	
Fracture location		<0.001
Supracondyle	73 (53.3)	
Lateral condyle	54 (39.4)	
Medial epicondyle	10 (7.3)	
Reduction technique		<0.001
Closed	101 (73.7)	
Open	36 (26.3)	

The Baumann angle had no statistically significant difference from pin removal day through 1-2 weeks and beyond 12 weeks (71.95 ± 3.77 vs 71.92 ± 3.84 vs 72.12 ± 3.76, p=0.368). Conversely, the lateral humeral-capitellar angle demonstrated a statistically significant difference across different time intervals. Analysis showed a slight decrease at beyond 12 weeks (51.44 ± 2.29 vs 51.37 ± 2.47 vs 50.92 ± 3.42, p=0.010) (Table 2).

**Table 2:** Baumann angle and lateral humeral-capitellar angle comparison Data represented in Mean ± SD

	Day 0 (Pin Removal Day)	1-2 weeks Follow-up	>12 weeks Follow-up	p-value
Baumann angle	71.95 ± 3.77	71.92 ± 3.84	72.12 ± 3.76	0.368
Lateral humeral-capitellar angle	51.44 ± 2.29	51.37 ± 2.47	50.92 ± 3.42	0.01

Anterior humeral line alignment was unchanged in all patients, with 60 cases (43.8%) demonstrating anterior one-third alignment and 77 cases (56.2%) demonstrating middle one-third (p=1.0). In contrast, callus formation showed a significant temporal progression toward four cortices (p<0.001). At the time of pin removal, 32 patients (23.4%) exhibited callus formation involving three cortices; however, all subsequently demonstrated progression within 1-2 weeks (Table 3).

**Table 3:** Anterior humeral line alignment and number of cortices with callus comparison Data represented in N (%)

	Day 0 (Pin Removal Day)	1-2 weeks Follow-up	>12 weeks Follow-up	p-value
					Anterior humeral line alignment				1
Anterior 1/3	60 (43.8)	60 (43.8)	60 (43.8)						
Middle 1/3	77 (56.2)	77 (56.2)	77 (56.2)						
Posterior 1/3	0 (0)	0 (0)	0 (0)						
Cortices with callus				<0.001
1	0	0	0	
2	0	0	0	
3	32 (23.4)	0	0	
4	105 (76.6)	137 (100)	137 (100)	

**Figure 1:**
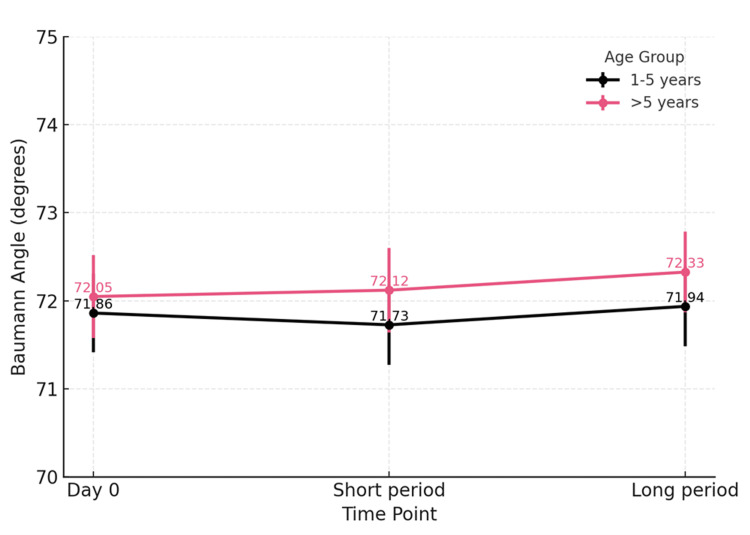
Subgroup analysis of age groups: Baumann angle

**Figure 2:**
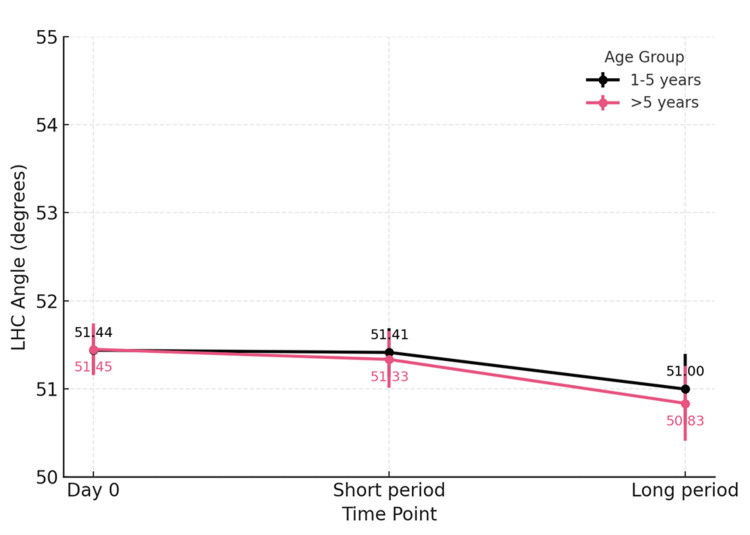
Subgroup analysis of age groups: Lateral humeral-capitellar angle LHC angle is the lateral humeral-capitellar angle

Subgroup analysis by age (1-5 vs >5 years old) demonstrated no significant difference in either the Baumann angle (p=0.603 vs p=0.475) or LHCA (p=0.146 vs p=0.071). Notably, the LHCA exhibited a trend toward reduction at beyond 12 weeks among patients older than five years old (Figure 1 and Figure 2).

**Figure 3:**
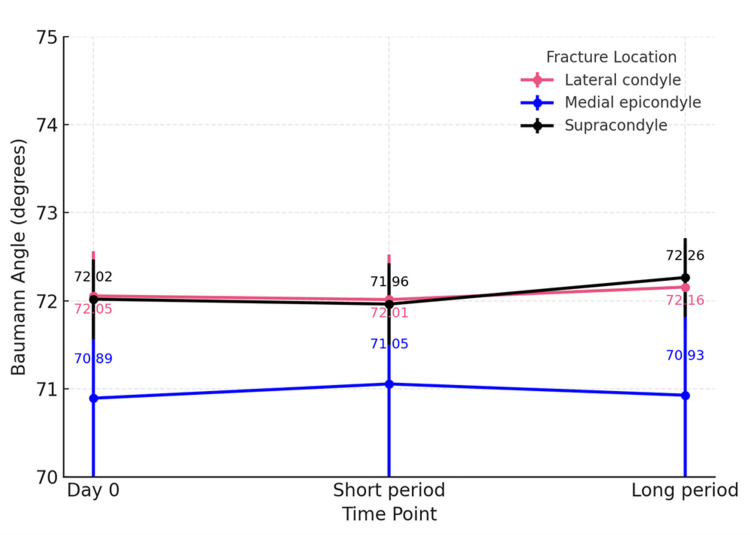
Subgroup analysis of fracture locations: Baumann angle

**Figure 4:**
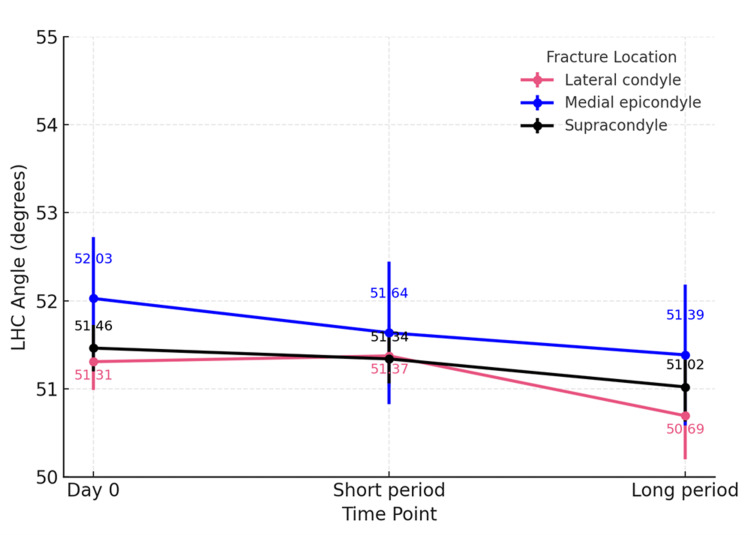
Subgroup analysis of fracture locations: Lateral humeral-capitellar angle LHC Angle is Lateral humeral-capitellar angle

Analysis by fracture locations (including supracondylar, lateral condylar, and medial epicondylar humeral fractures) revealed no significant change in the Baumann angle across groups (p=0.294, p=0.860 and p=0.964) (Figure 3). In contrast, the LHCA showed a statistically significant decrease at beyond 12 weeks in the lateral condyle subgroup (p=0.043), whereas patients with supracondylar and medial epicondylar fractures had a stable LHCA over time (p=0.2 and p=0.756) (Figure 4).

## Discussion

Displaced pediatric distal humeral fractures may still result in unfavorable outcomes, including cubitus varus or cubitus valgus deformities, even following surgical treatment [12,17]. Postoperative radiographs remained a routine follow-up management in practice for monitoring changes in alignment and bone union despite accumulating evidence demonstrating limited or no clinical utility. Ponce et al. evaluated 104 pediatric supracondylar fractures with closed reduction and pinning and found no significant changes in Baumann angle, lateral humeral-capitellar angle or lateral rotational percentage when comparing radiographs obtained within 10 days and beyond 10 days of pin placement [22]. Similarly in larger series, a recent ambi-directional cohort study of 1,512 pediatric supracondylar fractures reported that three-week postoperative radiographs altered management in only 0.4% of patients, all of whom required either extended immobilization or additional imaging. Importantly, no changes in treatment occurred among type II or type IV fractures, and only two cases (0.9%) of type III fractures were affected. Zhao et al. concluded that routine radiographs at the time of pin removal for intraoperatively stable fractures provide minimal clinical utility and increase financial burdens [28].

Even after pin removal, some surgeons still use radiographs for follow-up, although available evidence suggests these may be unnecessary. Karilius et al. also reviewed 572 pediatric supracondylar fractures treated with closed reduction and pinning and found that radiographs at the time of pin removal indicated the need for extended immobilization in only 20 cases (3.5%), with no additional changes to nonoperative management required at the final 6- to 8-week follow-up [21]. Zusman et al. found no change in management in 100 pediatric patients with supracondylar fractures treated with closed reduction and pinning at pin removal and at three weeks later. Therefore, follow-up radiographs after pinning may not provide clinical utility in the absence of other clinical findings [29]. We could not find any current articles regarding the role of follow-up radiographs for both lateral condylar and medial epicondylar fractures.

The aim of the present study was to evaluate the necessity of radiographs at different intervals after pin removal in pediatric distal humeral fractures that had undergone acceptable reduction and stable fixation. Specifically, we focused on radiographic changes in Baumann angle and lateral humeral-capitellar angle, both of which are key predictors of elbow deformity [25-27]. Assessments were performed at three postoperative time points: pin removal, short-term follow-up (1-2 weeks), and long-term follow-up (>12 weeks). We used beyond 12 weeks as a long-term follow-up period for nonunion with subsequent displacement of lateral condylar fractures usually diagnosed at this period [12].

Our findings demonstrated no statistically significant difference in the Baumann angle during either the short- or long-term follow-up periods. The lateral humeral-capitellar angle exhibited a slight decrease in long-term follow-up, particularly among patients older than five years old and those with lateral humeral condylar fractures, as identified in subgroup analyses. However, the LHCA values remained within the normal range in all cases. Additionally, all fractures demonstrated expected progression of callus formation to four cortices within short-term follow-up. Anterior humeral line alignment remained unchanged across all patients. Taken together, these results suggest that routine radiographs after pin removal are not warranted when acceptable reduction and stable fixation were achieved.

Strengths and limitations

This study included a relatively large cohort with extended follow-up, thereby providing robust insights into postoperative radiographic trends. However, potential selection bias may have arisen from surgeons’ discretion in operative protocols. Furthermore, although the Baumann angle and LHCA were the primary parameters analyzed, additional radiographic and clinical factors may contribute to long-term outcomes and warrant further investigation. 

## Conclusions

Follow-up radiographs demonstrated that the Baumann angle remained stable across pin removal day, short-term, and long-term, regardless of age or fracture location. In contrast, the LHCA showed a small decrease beyond 12 weeks after pin removal, most evident in patients older than five years and those with lateral humeral condylar fractures. Importantly, all angular measurements remained within the normal range, and complete bone union was achieved in every case. Our study indicated no role of routine postoperative radiographs after pin removal in displaced pediatric distal humeral fractures when acceptable reduction and stable fixation have been achieved intraoperatively.

## References

[1] Blumberg SM, Kunkov S, Crain EF, Goldman HS (2011). The predictive value of a normal radiographic anterior fat pad sign following elbow trauma in children. Pediatr Emerg Care.

[2] Robert EL, Ryan WS, Peter MW (1999). Pediatric elbow trauma. Orthoped Clin North Am.

[3] Baker M, Borland M (2011). Range of elbow movement as a predictor of bony injury in children. Emerg Med J.

[4] Ward WT, Rihn JA (2006). The impact of trauma in an urban pediatric orthopaedic practice. J Bone Joint Surg Am.

[5] Voth M, Lustenberger T, Auner B, Frank J, Marzi I (2017). What injuries should we expect in the emergency room?. Injury.

[6] Emery KH, Zingula SN, Anton CG, Salisbury SR, Tamai J (2016). Pediatric elbow fractures: a new angle on an old topic. Pediatr Radiol.

[7] Tomsan H, Grady MF, Ganley TJ, Nguyen JC (2021). Pediatric elbow: development, common pathologies, and imaging considerations. Semin Roentgenol.

[8] Tileston K, Frick SL (2020). Trash (the radiographic appearance seemed harmless) lesions about the elbow. Pediatric Orthopedic Trauma Case Atlas.

[9] Leitch KK, Kay RM, Femino JD, Tolo VT, Storer SK, Skaggs DL (2006). Treatment of multidirectionally unstable supracondylar humeral fractures in children. A modified Gartland type-IV fracture. J Bone Joint Surg Am.

[10] Song KS, Kang CH, Min BW, Bae KC, Cho CH, Lee JH (2008). Closed reduction and internal fixation of displaced unstable lateral condylar fractures of the humerus in children. J Bone Joint Surg Am.

[11] Mirsky EC, Karas EH, Weiner LS (1997). Lateral condyle fractures in children: evaluation of classification and treatment. J Orthop Trauma.

[12] Peter MW, David LS, John MF (2019). Rockwood & Wilkins' Fractures in Children. https://orthopaedics.lwwhealthlibrary.com/book.aspx?bookid=918.

[13] St. Clair JB, Schreiber VM (2019). Supracondylar humerus fractures. Oper Tech Orthopaed.

[14] Skaggs DL (1997). Elbow fractures in children: diagnosis and management. J Am Acad Orthop Surg.

[15] Garg S, Weller A, Larson AN (2014). Clinical characteristics of severe supracondylar humerus fractures in children. J Pediatr Orthop.

[16] Bashyal RK, Chu JY, Schoenecker PL, Dobbs MB, Luhmann SJ, Gordon JE (2009). Complications after pinning of supracondylar distal humerus fractures. J Pediatr Orthop.

[17] Pirone AM, Graham HK, Krajbich JI (1988). Management of displaced extension-type supracondylar fractures of the humerus in children. J Bone Joint Surg Am.

[18] Ott N, Hackl M, Leschinger T, Wegmann K, Müller LP (2020). Predictors of avascular necrosis of the trochlea after pediatric supracondylar humerus fractures. Obere Extremität.

[19] Sankar WN, Hebela NM, Skaggs DL, Flynn JM (2007). Loss of pin fixation in displaced supracondylar humeral fractures in children: causes and prevention. J Bone Joint Surg Am.

[20] Ip D, Tsang WL (2007). Medial humeral epicondylar fracture in children and adolescents. J Orthop Surg (Hong Kong).

[21] Karalius VP, Stanfield J, Ashley P (2017). The utility of routine postoperative radiographs after pinning of pediatric supracondylar humerus fractures. J Pediatr Orthop.

[22] Ponce BA, Hedequist DJ, Zurakowski D, Atkinson CC, Waters PM (2004). Complications and timing of follow-up after closed reduction and percutaneous pinning of supracondylar humerus fractures: follow-up after percutaneous pinning of supracondylar humerus fractures. J Pediatr Orthop.

[23] Karamitopoulos MS, Dean E, Littleton AG, Kruse R (2012). Postoperative radiographs after pinning of supracondylar humerus fractures: are they necessary?. J Pediatr Orthop.

[24] Williamson DM, Coates CJ, Miller RK, Cole WG (1992). Normal characteristics of the Baumann (humerocapitellar) angle: an aid in assessment of supracondylar fractures. J Pediatr Orthop.

[25] Silva M, Pandarinath R, Farng E, Park S, Caneda C, Fong YJ, Penman A (2010). Inter- and intra-observer reliability of the Baumann angle of the humerus in children with supracondylar humeral fractures. Int Orthop.

[26] Hasegawa M, Suzuki T, Kuroiwa T (2018). Reliability of radiographic measurement of lateral capitellohumeral angle in healthy children. Medicine (Baltimore).

[27] Simanovsky N, Lamdan R, Hiller N, Simanovsky N (2008). The measurements and standardization of humerocondylar angle in children. J Pediatr Orthop.

[28] Zhao G, Trottier ER, Ng K (2024). Eliminating rote postoperative radiographs for surgically managed pediatric supracondylar humerus fractures. Can J Surg.

[29] Zusman NL, Barney NA, Halsey MF, Yang S (2020). Utility of follow-up radiographs after pin removal in supracondylar humerus fractures: a retrospective cohort study. J Am Acad Orthop Surg.

